# A Novel Method for Verifying War Mortality while Estimating Iraqi Deaths for the Iran-Iraq War through Operation Desert Storm (1980-1993)

**DOI:** 10.1371/journal.pone.0164709

**Published:** 2016-10-21

**Authors:** Shang-Ju Li, Abraham Flaxman, Riyadh Lafta, Lindsay Galway, Tim K. Takaro, Gilbert Burnham, Amy Hagopian

**Affiliations:** 1 University of Washington School of Public Health, Department of Global Health, Seattle, Washington, United States of America; 2 Al-Mustansiriya University School of Medicine, Baghdad, Iraq; 3 Lakehead University, Thunder Bay, Ontario, Canada; 4 Simon Fraser University, Faculty of Health Sciences, Burnaby, BC, Canada; 5 Johns Hopkins Bloomberg School of Public Health, Baltimore, MD, United States of America; Laval University, CANADA

## Abstract

**Objectives:**

We estimated war-related Iraqi mortality for the period 1980 through 1993.

**Method:**

To test our hypothesis that deaths reported by siblings (even dating back several decades) would correspond with war events, we compared sibling mortality reports with the frequency of independent news reports about violent historic events. We used data from a survey of 4,287 adults in 2000 Iraqi households conducted in 2011. Interviewees reported on the status of their 24,759 siblings. Death rates were applied to population estimates, 1980 to 1993. News report data came from the ProQuest *New York Times* database.

**Results:**

About half of sibling-reported deaths across the study period were attributed to direct war-related injuries. The Iran-Iraq war led to nearly 200,000 adult deaths, and the 1990–1991 First Gulf War generated another approximately 40,000 deaths. Deaths during peace intervals before and after each war were significantly lower. We found a relationship between total sibling-reported deaths and the tally of war events across the period, p = 0.02.

**Conclusions:**

We report a novel method to verify the reliability of epidemiological (household survey) estimates of direct war-related injury mortality dating back several decades.

## Introduction

War and armed conflict significantly undermine the health of populations.[1–4] Wide-scale violence causes immediate casualties, but also creates long-term circumstances that undermine population health. Health care, transportation, water, sewage, electricity, communications and other infrastructure components are disrupted, while shifting resources towards the military.[5] To understand the effect of conflict on the health of populations, public health researchers need better methods to assess war-related morbidity and mortality for past events.

The conventional public health measure of war is mortality, although in settings where robust health information reporting persists despite war, other measures such as injury,[6] disability and mental health effects are also important to track.[7] Scientists have struggled to find accurate methods to count war-related deaths when normal civil registration systems are compromised. This is particularly true for past conflicts where estimates by war historians or journalists are commonly used. In this paper, we report a new approach to measuring historical war-related mortality that covers a 30-year period in Iraq, including the Iran-Iraq war, the First Gulf War (including both the Iraq invasion of Kuwait and the brief Coalition armed response), and the periods of relative peace bracketing those events.

The Iran-Iraq War was the longest conventional war of the 20^th^ Century, lasting 1980 to 1988.[8,9] This was followed by almost two years of calm before Iraq invaded Kuwait in early August, 1990[10] (the First Gulf War). Iraq occupied Kuwait for a few months until January, 1991, when U.S.-led Coalition forces briefly entered southern Iraq in “Operation Desert Storm.”[11] Coalition military action was primarily limited to aerial bombardment, and hostilities ceased in February 1991. Economic sanctions imposed under United Nations (UN) Security Council resolution 661 continued until May 2003.[12]

In the absence of civil registration and vital statistics systems, population sampling remains the standard method for estimating deaths from ongoing or recent conflicts. No population survey methods have attempted to estimate mortality during these earlier Iraq conflicts. To fill this gap, we analyzed data from a nationally representative cross-sectional household survey in 2011, using the sibling survival method.[13] To judge the validity of these methods with particular concern for the long recall period, we collected independent data from news and historical event reports to determine whether sibling death reports followed expected patterns in relation to changing intensity of war activity over time. Our research questions were: 1) *What was the war-related mortality (as reported by adult siblings) in Iraq for the 14-year period*, *1980 through 1993*, *which included the Iran-Iraq war and the First Gulf War?* And 2) *Can we verify this mortality estimate by analyzing the frequency of war-related news events for the corresponding time period?*

## Methods

We had review board approval from each participating institution in the study, including University of Washington, Simon Fraser University, and Johns Hopkins University. Methods complied with the guidelines for epidemiological research set out by the Council for International Organizations of Medical Sciences. An ethicist experienced in international research associated with the Institute of Translational Health Sciences at the University of Washington, Benjamin Wilfond, further reviewed the protocols participant and interviewer protection. The participants provided their verbal informed consent in this study. The reason we chose verbal consent instead of written consent is 1) The subjects only needed to answer the question listed on approved protocol; 2) The safety concern. This research had been conducted in the certain post-conflict area. The subjects may not provide their written form information. The interview will be only after obtaining verbal consent following our human subjects protocol. We didn’t record participant consent. The ethics committees/IRBs approved this consent procedure.

### Sibling questionnaire

The University Collaborative Iraq Mortality Study conducted a nationally representative cross-sectional survey of all adults living in 2,000 households in 100 randomly selected clusters across Iraq in mid-2011.[14] Data collectors traveled to 100 geographically independent neighborhood clusters across Iraq (randomly selected, with the probability of selection proportional to estimated population size). Cluster selection relied on LandScan^TM^, a commercial spatial population dataset, and Google Earth^TM^ imagery (details in Galway et al, 2012[15]). Google Earth^TM^ imagery was used to randomly select an index (start) house in each cluster, and we employed protocols to select the 19 nearby homes. All adults in the 20 homes in the 100 clusters were interviewed about all their siblings. We removed duplicated family siblings (when two siblings could have reported on the same family) by instructing surveyors to report results only from the sibling whose birthday was nearest to the date of our visit.

In each household, we interviewed each adult and recorded the current vital status of the sibling (alive or dead), year of birth and sex of sibling (See S1 File). If the sibling was dead, we recorded cause of death, year of death, and location of death. If a death was from direct war-related injury, we asked about specific mechanism of death (explosion, gunshot, etc.), and who the suspected perpetrator was (Iraqi army, Coalition force, criminals, etc.).

Table 1 reflects the sibling respondents to our survey by governorate, an administrative unit similar to province, with numbers of siblings and reported deaths. Detailed information about the household survey and the mortality associated with the 2003 Coalition invasion are reported in Hagopian *et al*, 2013.[14]

**Table 1:** Sample size and counts of siblings in Iraq, by governorate.

Governorate	Number of Clusters	Proportion of Sample	Number of Adults Reporting on Siblings	Number of Unique Siblings Reported	Number of Siblings Reported dead in 1980–1993	Percentage of Siblings Missing Cause of Death
**Al-Anbar**	7	0.07	375	2,540	66	10.61%
**Al-Basrah**	8	0.08	335	1,908	57	15.79%
**Al-Muthanna**	1	0.01	61	371	18	5.56%
**Al-Najaf**	2	0.02	68	398	14	0.00%
**Al-Qadisiya**	3	0.04	234	1,366	54	7.41%
**Al-Sulaimaniya**	7	0.07	291	1,822	87	2.30%
**Babylon**	3	0.03	154	877	34	8.82%
**Baghdad**	23	0.23	1066	5,442	168	4.76%
**Diala**	5	0.05	183	1,050	29	0.00%
**Duhouk**	2	0.02	98	606	26	0.00%
**Erbil**	9	0.09	357	2,055	55	3.64%
**Kerbela**	2	0.02	109	607	15	40.00%
**Kirkuk**	2	0.02	98	502	20	5.00%
**Maysan**	3	0.03	118	704	26	3.85%
**Ninevah**	12	0.13	437	2,658	57	10.53%
**Salah Al-Deen**	3	0.03	111	669	19	5.26%
**Thi Qar**	2	0.03	72	454	16	0.00%
**Wasit**	3	0.03	120	730	21	0.00%
**All governorates**	98	1.00	4,287	24,759	782	6.52%

**Source of data:** Data provided by the University Collaborative Iraq Mortality Study, which collected household and sibling data from 2000 households across Iraq in 2011. Table 1 excludes data from 2 dropped clusters. Table also includes all sibling deaths, regardless of age.

### Population data to calculate rates

We calculated rates of death from our sample (deaths divided by total sibling population), and multiplied these by total national population numbers (categorized by age and sex intervals) to derive total population deaths by demographic category. Population data for the years of this study came from the UN Department of Economic and Social Affairs, Population Division.[16] These deaths were summed across categories to derive a total adult death count for each year, 1979–1993. We report the data as a weekly death rate.

Our summary of adult mortality is *45q15*, which is the risk that an individual reaching 15 years of age will die before his or her 60th birthday. Male *45q15* mortality risks range from below 0.05 in a few countries to above 0.45 in a handful of high-mortality African nations.[17]

While the majority of sibling reports in our survey included the month of death, many death events (40%) were only available at the annual level. If month was missing, we assumed the month of June. This could have affected results, as, for example, the Iran-Iraq war started in September of 1980.

### Mortality-related events data base

To answer our second research question, we collected information about events associated with Iraq and Iran for the time period under study. We reviewed “ProQuest Historical Newspapers: *The New York Times*,”[18] because it had a comprehensive collection of *New York Times* articles for the years of this study. Our search was limited to the period 1980 through 1993, limiting the search to “Iraq” or “Iran.” In the field “document type,” we limited the search to “Article,” “Banner,” “Editorial,” “Front page article,” “Other,” “Military war news,” and “Review.” We excluded “Legal notice,” “Lottery numbers,” “Marriage,” “Obituary,” “Photo standalone,” “Masthead,” “Real estate transaction,” “Soldier list,” “Stock quote,” “Table of contents,” and “Weather.”

The goal was to identify independent war events in each country that could have influenced Iraqi mortality. Our search produced 31,357 articles. In reviewing each article, and after eliminating reports of duplicate events, we counted 4,769 unique events. Each of these events was cataloged by month, year, location, type, and parties reported to be involved. To catalogue locations, we divided events between Iran and Iraq. Those that occurred in Iraq were identified by governorate. For imprecise locations, a regional classification was assigned.

Events were also categorized into 11 types: war-related, including weapons mobilization, troop mobilization and armed conflict, violent political unrest, hostage-taking, humanitarian intervention, domestic political event, elections, cross-national diplomatic events, policies or actions regarding the extraction of natural resources (e.g., oil or natural gas), financial news, and natural disasters.

### Study hypothesis

We hypothesized that adults in the Iraq study households could, in 2011, report details of sibling deaths even when deaths occurred 30 or more years ago. To assess this hypothesis, we reviewed independent reports of war and other mortality-related events in media reports over the period. We also hypothesized that during periods when the media reported intense violence and deaths, reports of deaths among siblings of householders interviewed would be elevated.

### Analysis

Estimates of mortality using the sibling survival method are subject to predictable biases.[19–21] Sibships that experience a higher mortality risk are underrepresented in surveys, because these siblings are less likely to survive to be able to report (survival bias). Additionally, larger sibships are overrepresented, because there are more siblings in the sampling frame. We used current methods to adjust for these biases.[13] Briefly, we estimated the number of likely missing sibling deaths from the sample by age and sibship size through iteration for sibship sizes of one and two. We then added back these missing siblings to the observed sample before calculating final age-specific mortality rates.

We calculated uncertainty intervals (UIs), which can be interpreted similarly to confidence intervals, at the 95% level for crude death rates for each time period using a bootstrapping method. We randomly selected 1000 times from our original sibling survival spreadsheet, where every row of data (each sibling) had a chance to be reselected with the same time period setting. For each of these 1,000 replicates, we calculated death rates for the same time period. The 2.5^th^ and 97.5^th^ percentiles of these 1,000 values served as our lower and upper bounds, respectively.[22,23]

To test the hypothesis that sibling death reports would be associated with the number of reported news events, we conducted linear regression analysis to determine the association between number of news events and number of reported sibling deaths. We compared total events to total deaths, war events to total deaths, total events to war deaths, and war events to war deaths in separate regression analyses. For each regression, we calculated a coefficient to assess the slope of the best-fit line between events and deaths. Each regression also produced a Spearman correlation and P-value.

For statistical analysis, we used Python Language Reference, version 2.7, Python Software Foundation, available at python.org.

### Ethics approvals

An ethical review board from each participating institution reviewed the collection of sibling death data in the original University Collaborative Iraq Mortality Study household survey and provided clearances.[14]

## Results

### Sibling survey results and national extrapolations

We interviewed 4,287 adult Iraqis in 1,960 households who reported on the vital status (alive or dead) of 24,759 siblings. (We dropped 2 clusters of 20 households each for technical reasons, see original manuscript.[14]) On average, siblings reported having 5.8 total brothers and/or sisters. The average adult reported 0.18 dead siblings (n = 782 dead) between 1980 and 1993. Of the total dead, 76.5% were male (n = 599). Most siblings were able to report a cause of death for their deceased family member (94%). In applying the zero-survivor correction, 7.34 missing siblings were added to the data set after three iterations. See Table 1.

**Fig 1:**
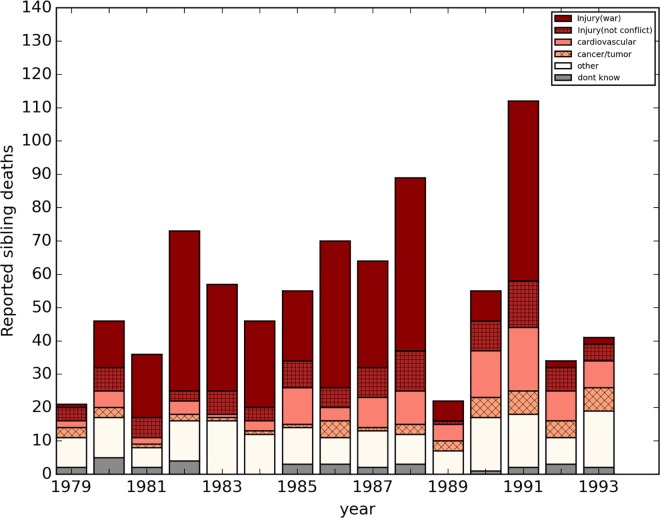
Raw number of siblings death by year and cause, Iraq 1979–1993. **Source of data:** Survey of 4,287 adults in 1,960 households in Iraq between May and July of 2011,782 sibling deaths by cause between 1980 and 1993. Data provided by the University Collaborative Iraq Mortality Study, which collected household and sibling data from 2000 households across Iraq in 2011. Direct war-related injury deaths / total deaths = 45.3%. Among other deaths, 23.1% were attributed to cardiovascular disease, 22.2% to other injury, 10.7% to cancer, 37.0% to “other” and 7.0% to “don’t know.”

Total deaths by cause and year are shown in Fig 1. First, we applied death rates calculated from our sample of Iraqi adults to the age-, time-, and sex-specific UN population estimate totals.[16] Then we aggregated the number of deaths among adults aged 15–60 years, between 1980 and 1993. Deaths in the age range totaled nearly half a million (498,761), (95% UI 418,362 to 583,414). Direct war-related injury mortality comprised almost half (48%) of total deaths over the period.

**Fig 2:**
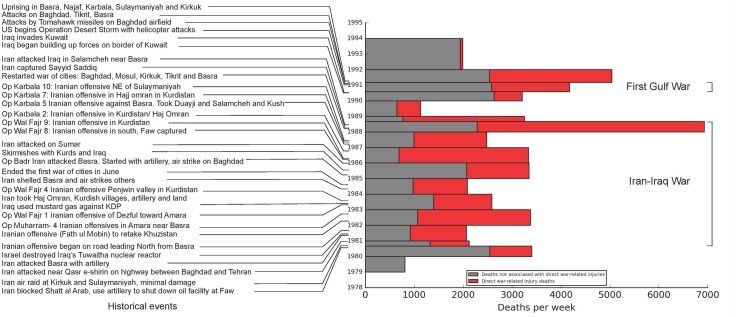
Estimates of numbers of adult deaths per week in Iraq, 1979–1993, by cause as reported by siblings. **Source of mortality data:** Survey of 4,287 adults in 1,960 households in Iraq between May and July of 2011, reporting on 782 sibling deaths between 1980 and 1993. Data provided by the University Collaborative Iraq Mortality Study, which collected household and sibling data from 2000 households across Iraq in 2011; Event calendar collected from literature review. [24–27]

Total deaths per week in relation to war events are reported in Fig 2. The Iran-Iraq conflict (Sept. 1980 through Sept. 1988) produced an estimated 174,289 adult (ages 15–60) direct war-related injury deaths, according to calculations based on our sibling reports (95% UI 132,213 to 215,576). During the interval between the two wars (December 1988 through August 1990), we estimated 19,800 Iraqi direct war-related injury deaths (95% UI 5,591 to 38,002).

War-related injury deaths directly associated with the Gulf War totaled 11,187 (over a six-month period, 95% UI 3,797 to 19,512). An additional 25,036 war-related deaths were reported during the eight-month period immediately following the Coalition withdrawal (95% UI 14,048 to 44,953), presumably as the result of injuries that eventually resulted in death, but other reasons (war and non-war related) could pertain. 1,278 Gulf War-related injury deaths also were reported between 1992 and 1993. (95% UI 0 to 4,561).

**Fig 3:**
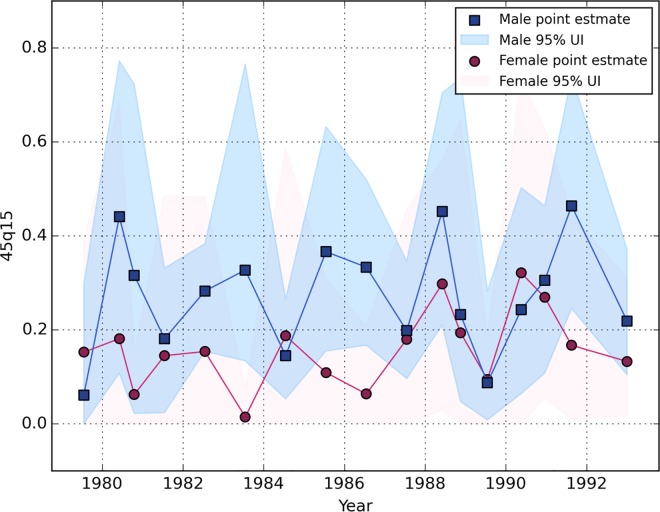
Estimates of the probability of dying between age 15 and age 60, in Iraq 1979–1993. **Source of data:** Survey of 4,287 adults in 1,960 households in Iraq between May and July of 2011, reporting on 782 sibling deaths by cause between 1980 and 1993. Data provided by the University Collaborative Iraq Mortality Study, which collected household and sibling data from 2000 households across Iraq in 2011. The blue points illustrate the estimation of the male 45q15, and the light blue shading illustrates the male 95% uncertainty intervals; The red points illustrate the estimate of female 45q15, and the light red shading illustrates the female uncertainty intervals.

Fig 3 reports the probability of death before age 60, by sex, for those who had lived to age 15 years in Iraq (*45q15*). Female death reports rose to their highest levels during the War of Cities (1988) and the period just prior to the invasion of Kuwait (1990). Male and female mortality reports were identical during the peace period between the end of the Iran-Iraq war and the start of the Kuwait war.

Iran-Iraq direct war-related injuries resulting in mortality were reported by siblings to be largely attributable to gunshots (41%) and airstrikes (17%). Similarly, the largest portions of First Gulf War injuries were also attributed to gunshots (31%) and airstrikes (26%). Most adult siblings did not name a particular responsible party (e.g., Iraqi forces or Iranian forces) for these deaths. See Table 2.

**Table 2:** Counts of reported violent deaths in Iraq by responsible party and by cause, by war and non-conflict intervals.

Sub-category	Time period				Total count	Percentage for 1980–1993
	Iran-Iraq war	Peaceful interval	First Gulf war	Aftermath		
	1980–1988	1989	1990–1991	1992–1993		
**Responsible part for violent deaths (Source: household reports)**						
**Coalition forces**	1	0	19	0	20	5.67%
**Militia**	2	1	6	2	11	3.12%
**Criminals**	2	1	1	0	4	1.13%
**Iraq army**	25	1	17	0	43	12.18%
**Iraq police**	36	1	11	0	48	13.60%
**Other/unknown**	215	2	9	2	228	64.31%
**All responsible parties**	281	6	63	4	354	100%
**Cause of violent deaths (Source: household reports)**						
**Gunshot**	117	3	20	1	141	39.66%
**Car bomb**	0	1	1	0	2	0.57%
**Airstrike**	50	1	17	1	69	19.55%
**Road accident**	4	0	1	0	5	1.42%
**Other explosion**	25	0	3	0	28	7.93%
**Other war/don’t know**	85	1	21	2	109	30.88%
**All cause**	281	6	63	4	354	100%

**Source of data:** Survey of 4,287 adults in 1,960 households in Iraq between May and July of 2011. Data provided by the University Collaborative Iraq Mortality Study, which collected household and sibling data from 2000 households across Iraq in 2011.[14,15]

Of deaths *not* attributable to war-related injuries during the entire time period (1979–1993), nearly one in four (23%) were attributable to unintentional injuries (e.g., car crashes or falls) and a similar number (24%) were cardiovascular in nature. About 11% were cancer related, and another 11% were neonatal. See Fig 1.

### News event tabulations

We identified 4,769 independent, potentially war-related, news events from among the 31,357 articles produced by the ProQuest database search terms of *New York Times* stories.[18]

**Fig 4:**
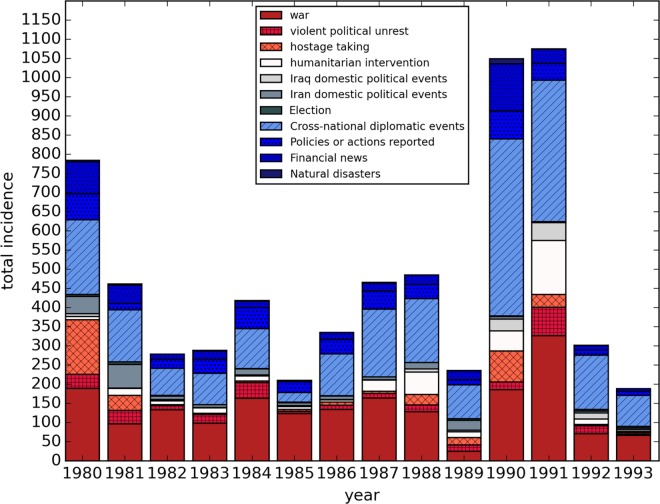
Number of *New York Times* news stories by year, 1980–1993, about Iraq and/or Iran. **Source of data:** Our review of ProQuest Historical Newspapers database for NY Times articles about Iran or Iraq for the period 1980–1993. Of 31,357 articles produced, this analysis displays results for 4,769 non-duplicate events by type.

We coded 1,905 (40.5%) of events as directly war-related (including weapons mobilization, troop mobilization and armed conflict). We coded 329 events as violent political unrest, hostage taking, humanitarian intervention, or domestic political events in Iraq. We counted 30 reports of election-related events in Iraq, and another 2,206 diplomatic events. Another 507 events related to policies or actions regarding the extraction of natural resources (e.g., oil or natural gas). There were 449 news events related to financial issues, and another 28 related to natural disasters. Across all events, we identified 52 separate nations or organizations associated with events. About 52% were associated with Iraq and 54% with Iran (a story could be tagged as one or the other or both), 22% were associated with the United States, and 10% with the United Nations. We coded only one or two primary actors for each event. Raw events data are reported by cause and year in Fig 4.

### Correlation between news event counts and direct war-related injury deaths

We hypothesized that sibling deaths reported in 2011 would be reliable, despite long recall periods for deaths at the start of our study period.

We analyzed mortality in relation to total news reports between 1980 and 1993 in four ways:

- Total news events in relation to the point estimates of total deaths;
- Total news events in relation to the point estimates of direct war-related injury deaths;
- War-related news events in relation to the point estimates of total death; and
- War-related news events in relation to the point estimates of direct war-related injury deaths.

**Fig 5:**
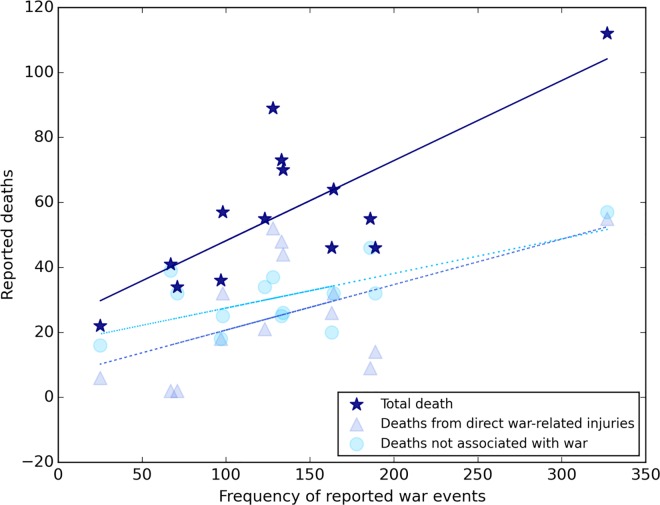
Correlation between reported total Iraqi sibling deaths and new-reported war events, 1980–1993. **Source of data:** Deaths come from a survey of 4,287 adults in 1,960 households in Iraq between May and July of 2011, reporting on 782 sibling deaths by cause between 1980 and 1993. Data provided by the University Collaborative Iraq Mortality Study, which collected household and sibling data from 2000 households across Iraq in 2011. Total incidents come from our review of ProQuest Historical Newspapers database for *New York Times* articles about Iran or Iraq for the period 1980–1993, illustrating 4,769 non-duplicate events. Spearman correlation between war events and total sibling-estimated deaths is 0.60, *P-value* 0.02 (note: sensitivity analysis removing strong upper right hand data point, associated with Desert Storm, reduces certainty to p-value 0.07). Spearman correlation between war events and violent war deaths is 0.51, *P-value* 0.06. Spearman correlation between war events and deaths not associated with war is 0.41, *P-value* 0.14.

Of the four comparisons, only one was statistically significant (p = 0.02, Spearman correlation 0.61): *war-related* news events were associated with *total* sibling-reported deaths. Fig 5 illustrates the relationship between war events (as a subset of total events) and sibling-reported deaths (with separate lines for total, war-related, and non-war related deaths). We conclude that an increase in war-related news reports corresponded with an increase in sibling reports of deaths for the same period of time.

## Discussion

A goal of this study was to improve on existing methods to estimate war deaths, with a focus on the Iran-Iraq war (1980–1988) and the Gulf War (1990–1991). Such methods would have important implications for estimating deaths for other conflicts within living memory. We were able to verify our sibling survey estimates of war-related mortality for the periods of interest using our proposed method (news event counts); further, our counts were in sync with previous historical methods.

### Iran-Iraq war

The origins of the Iran-Iraq war, which began when Iraq invaded Iran in September 1980, were complex. The war ignited after a series of border disputes, fueled by hegemonic aspirations and religious and cultural divides. Iraq feared the Iranian Revolution in 1979 could mobilize Iraq’s long-suppressed Shia majority. Territorial disputes involved the Shatt al-Arab waterway and control of resources.[24–27] Western countries have long had strategic interests in the region, and alternately supported either Iran or Iraq, further fueling the tensions.[25,27] A variety of high intensity weapons were used, including chemical agents.[28,29]

The Iraqi adult siblings in our study reported deaths in a pattern that coincides to a large extent with the pattern of war-related events during the period, which we detail in Fig 2. Prior to the Iraq invasion of Iran in 1980, death reports were at a relatively low level. Both total deaths and direct war-related injury deaths grew in the years immediately following the invasion. An on-again, off-again ceasefire arrangement was established in 1987, after which death reports fell again. We measured the greatest number of deaths per week in early 1988, during the fifth “war of the cities.” In 1989, a year with no declared war, deaths from our sibling survey were at their lowest point since the beginning of our study period. When war-related deaths occurred during years of peace, we presume deaths are related to injuries sustained earlier when war was ongoing, delayed reporting, conversion of missing persons to death status, or inaccuracies with year of report.

Analyst Dilip cites conservatives estimates of 367,000 deaths (along with 700,000 injuries) in the Iran-Iraq war.[30] Of these deaths, 105,000 were Iraqis.[30] Author Rob Johnson cites 200,000 Iraqis deaths, 400,000 wounded and 70,000 taken prisoner during the 8-year war.[26] Our sibling data produced estimates of 175,000 adult Iraqi deaths between age 15 and 60 attributable to direct war-related injuries sustained, and another 19,800 in the immediate post-war period. Our estimates are roughly in line with Hiro and Johnson numbers, although somewhat higher when considering our estimates are only for adults.

Iranian civil registration records recorded 183,623 deaths associated with the 1980–1988 Iran-Iraq war.[31]

### First Gulf War

After the Iran-Iraq war, armed conflict and sanctions conspired to elevate mortality, migration and misery.[32] Iraq’s gross domestic product (GDP) dropped to $245 million from $66 billion in 1989, and estimates of infrastructure damage totaled $170 billion.[32,33] Vulnerable populations suffered food scarcity.[34,35] Kuwaiti decisions to escalate oil production drove a drop in oil prices, fueling tensions with Iraq. Deaths rose in 1990, as Saddam Hussein mobilized for the invasion of Kuwait in August. Iraq invaded Kuwait in August, 1990, 23 months after the Iran-Iraq war ended.[36]

“Operation Desert Storm” Coalition forces attacked Iraq in January of 1991, at which time sibling death reports more than doubled. Following Desert Storm, direct war-related injury deaths dropped to low levels. Our analysis ended before cumulative effects of sanctions could be counted.[37–41] Even though the period of Desert Storm was short (mid-1990 through early 1991), the adult (age 15–60) deaths associated with it substantially exceeded the highest death rates associated with the city bombing campaigns in the Iran-Iraq war. As with the Iran-Iraq war, direct war-related injury deaths persisted in the months following the official end of the conflict, likely for similar reasons.

For the First Gulf War (August 1990 to February 1991), death estimates have varied widely. A U.S. Census demographer, Beth Osborne Daponte, estimated 49,000–63,000 military deaths plus 3,500 civilian deaths from direct war effects.[4] A military analyst, Trevor DuPuy, estimated about half that. Former U.S. Attorney General Ramsey Clark estimated 25,000 Iraqi civilians died during the Coalition bombings of southern Iraq. Another 25,000 died soon thereafter, which Clark attributed to damaged infrastructure and other disruptions.[42] Our sibling survey allowed us to estimate nearly 80,000 total adult deaths during the First Gulf War time period, with just fewer than half attributable to direct war-related injuries. Given the wide range of uncertainty for estimates associated with this conflict, Daponte’s and Clark’s estimates are in line with ours.

While we have no primary data in our study to measure Kuwaiti deaths in the First Gulf War, other sources have estimated about 3,000 Kuwaiti lives were lost.[43]

### Death patterns

An unexpected finding was that direct war-related injury deaths appeared to increase in the months just before the start of both wars. We offer three possible explanations. One is the artifact of missing month of death (when missing a month, we set the death to June; the Iraq-Iraq war started in September, and the Gulf War started in August). A second is that the mobilization for war itself produces violent deaths. Another possibility is a margin of error in people’s ability to recall time of death that expands over a several month period, resulting in simple measurement error. We did not identify published literature addressing this issue, suggesting a possible area for future research.

This study has other limitations. Ideally, sibling survival method calculations require information from those interviewed on the month, year and cause of death. Recall bias is a major threat to accuracy. Helleringer *et al* improved the sibling survival method with improved interviewer training, the use of a historical events calendar, and allowing interviewees free recall with interviewer prompts.[21] Our interviewers used an events calendar with widely known events for reference points (See S2 File). We used the most recent recommendations for adjusting calculations to the method, resulting in only a modest 7.34 missing siblings for our zero-survivor correction. Because family size is large in Iraq, fewer corrections were required for small family size.[44,45]

Immigration bias is most certainly another source of possible undercount. In the period leading up to the two wars, Iraq had a large migrant labor force, both internally and third-country nationals, the latter not likely having families still resident in Iraq. Another limitation is that in our regression analysis to search for a relationship between death estimates and the number of reported war events we used point estimates rather than attempting to account for the uncertainty range around those estimates.

According to the UN, approximately 900,000 Iraqis left their country between 1980 and 1993.[16] Because some of these immigrants would have left few siblings behind to report, deaths among this group could not be counted.[32,44] Further, these estimates depend on census data, which is believed to be fairly accurate through 1987, but may have inaccuracies. The *New York Times* news reports may also be not be fully representative of events associated with mortality.

Our use of event reports in the media as an indicator of war intensity introduces a novel verification scheme. We believe this innovation shows promise in settings where a conflict receives extensive media coverage, though less so where there is a lack of international interest. We found total sibling deaths increased in correlation with the count of war-related news reports.

Nearly half of adult deaths across the study period were attributed to direct war-related injury mortality. During the two intervals of peace, siblings reported the lowest mortality figures. We conclude the Iran-Iraq war led to nearly 200,000 direct war-related adult injury deaths, and the First Gulf War generated another 40,000 deaths. Of total adult deaths during the Iran-Iraq war, nearly 60 percent were attributable to war-related injuries. We have contributed a novel research method to verify traditional epidemiological methods for estimating direct war-related injury mortality dating back several decades.

## Supporting Information

S1 File Survey questionnaire. (DOCX)

S2 File History calendar. (DOC)
